# Comparison of conformal and intensity modulated radiation therapy techniques for treatment of pelvic tumors. Analysis of acute toxicity

**DOI:** 10.1186/1748-717X-5-117

**Published:** 2010-12-14

**Authors:** Robson Ferrigno, Adriana Santos, Lidiane C Martins, Eduardo Weltman, Michael J Chen, Roberto Sakuraba, Cleverson P Lopes, José C Cruz

**Affiliations:** 1 Department of Radiation Oncology, Hospital Israelita Albert Einstein. Av. Albert Einstein, 627, São Paulo - SP - 05651-901 - Brazil; 2 Service of Dosimetry, Hospital Israelita Albert Einstein. Av. Albert Einstein, 627, São Paulo - SP - 05651-901 - Brazil; 3 Department of Medical Physics, Hospital Israelita Albert Einstein. Av. Albert Einstein, 627, São Paulo - SP - 05651-901 - Brazil

## Abstract

**Background:**

This retrospective analysis reports on the comparative outcome of acute gastrointestinal (GI) and genitourinary (GU) toxicities between conformal radiation therapy (CRT) and intensity modulated radiation therapy (IMRT) techniques in the treatment of patients with pelvic tumors.

**Methods:**

From January 2002 to December 2008, 69 patients with pelvic tumors underwent whole pelvic CRT and 65 underwent whole pelvic IMRT to treat pelvic lymph nodes and primary tumor regions. Total dose to the whole pelvis ranged from 50 to 50.4 Gy in 25 to 28 daily fractions. Chemotherapy (CT) regimen, when employed, was based upon primary tumor. Acute GI and GU toxicities were graded by RTOG/EORTC acute radiation morbidity criteria.

**Results:**

Absence of GI symptoms during radiotherapy (grade 0) was more frequently observed in the IMRT group (43.1% versus 8.7; *p *< 0.001) and medication for diarrhea (Grade 2) was more frequently used in the CRT group (65.2% versus 38.5%; *p *= 0.002). Acute GI grade 1 and 3 side effects incidence was similar in both groups (18.5% versus 18.8%; *p *= 0.95 and 0% versus 7.2%; *p *= 0.058, respectively). Incidence of GU toxicity was similar in both groups (grade 0: 61.5% versus 66.6%, *p *= 0.54; grade 1: 20% versus 8.7%, *p *= 0.06; grade 2: 18.5% versus 23.5%, *p *= 0.50 and grade 3: 0% versus 1.5%, *p *> 0.99).

**Conclusions:**

This comparative case series shows less grade 2 acute GI toxicity in patients treated with whole pelvic IMRT in comparison with those treated with CRT. Incidence of acute GU toxicity was similar in both groups.

## Background

Radiation therapy (RT) plays an important role in the treatment of malignant pelvic tumors, such as endometrial, cervical, rectal, vesical, and anal cancers. The use of the Intensity Modulated Radiation Therapy (IMRT) for treatment of these tumors has increased in the last years due to its capacity to decrease the amount of radiation dose delivered to the adjacent normal tissues, such as small bowel, bladder, rectum and bone marrow. Therefore, an advantage of this technique may be a potential benefit to decrease acute and late toxicities.

Gastrointestinal (GI) complications are among the most common undesirable side effects for patients treated with whole pelvic RT [1-3]. Diarrhea, a very frequent symptom, is not only uncomfortable but can also cause dehydration and nutrients malabsorption [4]. Genitourinary (GU) and hematological side effects are also relevant toxicities in the treatment of whole pelvis with RT. Several dosimetric studies have already shown significant reduction of radiation dose delivered to the small bowel, bladder, rectum, bone marrow and others organs-at-risk (OAR) with the use of IMRT rather than conventional or conformal radiotherapy (CRT) [5-15]. IMRT dosimetric characteristics provide a strong potential to reduce both acute and chronic RT toxicities. Published clinical outcomes with pelvic IMRT report reduced GI, GU and hematological toxicities when compared with conventional or CRT techniques but most of these studies are comparative case series or retrospective analyses with a small number of patients or with considerable heterogeneity [16-25].

This retrospective and comparative case series aimed to report results of acute GI and GU toxicities in patients with pelvic tumors treated with CRT versus IMRT techniques. This is the first clinical report on IMRT from South America. All other series are from United States of America (USA) and Europe.

## Methods

### Patients

We retrospectively compared 69 patients with pelvic tumors treated by whole pelvic CRT with 65 treated by whole pelvic IMRT, to evaluate the incidence and severity of acute GI and GU toxicities during the treatment. No patient had any symptom or morbidity before the RT treatment. Patients from both groups were treated between January 2002 and December 2008 in the Department of Radiation Oncology at the Hospital Israelita Albert Einstein, in São Paulo. Primary tumor sites included endometrium, cervix, rectum and anal canal in the CRT group and endometrium, cervix, rectum, anal canal and bladder in the IMRT group. Table 1 summarizes patients' characteristics of both groups.

**Table 1:** Characteristics of IMRT and CRT patients.

Characteristic	IMRT	CRT	*P *value
Patient number	65	69	0,53*

Age (y)			
Median	62	64	0.75*
Range	35 - 96	28 - 88	

Tumor site			
Endometrium	17 (26.1%)	20 (29%)	
Cervix	8 (12.3%)	3 (4.3%)	<0.001
Rectum	21 (32.3%)	40 (58%)	
Anal Canal	7 (10.8%)	6 (8.7%)	
Bladder	12 (18.5%)	0	

RT goal			
Adjuvant	29	39	0.004
Neo-adjuvant	14	23	
Definitive	22	7	

Gender			
Female	44	39	0.18
Male	21	30	

Chemotherapy			0.42
Yes	39	46	
No	26	23	

* Student t test

#### Radiotherapy

Patients from both groups were treated by whole pelvic RT following the International Commission on Radiation Units and Measurements (ICRU) No. 50 recommendations [26]. The clinical target volume (CTV) was defined as pelvic lymph nodes and primary tumor region and was contoured on individual axial CT slices. The lymph node regions were determined by encompassing the blood vessels with a 2 cm margin and based upon primary tumor site. The planning target volume (PTV) was created expanding the CTV by 1 cm. The small bowel region was defined by contouring the peritoneal cavity from the L4 level and excluding the rectum, bladder and blood vessels. The dose prescribed, to encompass at least 95% of the PTV, ranged from 45 to 50.4 Gy, delivered in 25 to 28 daily fractions in the phase of elective pelvic lymph node treatment. Treatment plannings were generated using the Eclipse Helios software (Varian Medical Systems, Palo Alto, CA) for CRT and IMRT. Dose volume restrictions used for OARs in both groups are described in Table 2.

**Table 2:** Dose volume restrictions for pelvic OARs used in Hospital Israelita Albert Einstein.

OAR	DOSE VOLUME RESTRICTIONS
**RECTUM**	≤ 55%: ≥ 47 Gy ≤ 40%: ≥ 65 Gy
	≤ 25%: ≥ 70 Gy ≤ 10%: ≥ 75 Gy
	Dmax: 82 Gy

**SMALL BOWEL**	≤ 100%: ≥ 40 Gy ≤ 66%: ≥ 45 Gy
	≤ 33%: ≥ 50 Gy
	Dmax: 60 Gy

**BLADDER**	≤ 55%: ≥ 47 Gy ≤ 30%: ≥ 70 Gy
	Dmax: 82 Gy

**FEMORAL HEAD**	Dmax: 50 Gy

Dmax: Maximum point dose.

In the CRT group, plans were based on 3 or 4 pelvic isocentric conformed coplanar fields with energy of 18-MV and patients were treated with a Varian CL2100 C linear accelerator (Varian Medical Systems, Palo Alto, CA) equipped with 80-leaf multileaf collimator, while in the IMRT group, treatment plannings were based upon a dynamic technique ("sliding window"), using 5 to 9 isocentric coplanar fields, equally spaced, with energy of 15-MV and patients were treated with Varian CL2300 EX linear accelerator (Varian Medical Systems, Palo Alto, CA) equipped with 120-leaf multileaf collimator.

#### Chemotherapy

Chemotherapy (CT), when employed, was based on primary tumor site. In both groups, the proportion of patients treated with CRT during the course of RT was equally balanced (Table 1). No patient with endometrium cancer was treated with CT, patients with cervix cancer, when treated with concomitant CT and RT, received weekly Cisplatin (40 mg/m^2^). Those with rectal cancer received oral daily Capecitabine (825 mg/m^2 ^BID, 5 days/week), those with anal canal cancer received 5-Flourouracil (1000 mg/m^2 ^continuous infusion days 1 - 4) and Mitomycin-C (10 mg/m^2 ^on day 1) during the first and last week of RT, and those with bladder cancer received weekly Cisplatin (40 mg/m^2^).

In the CRT group the proportion of patients who underwent CT according to the primary tumor site was: endometrium: 0/20 (0%); cervix: 1/3 (33%); rectum: 39/40 (98%) and anal canal: 6/6 (100%), while in the IMRT group the proportion was: endometrium: 0/17 (0%); cervix: 4/8 (50%); rectum: 18/21 (86%); anal canal: 7/7 (100%) and bladder: 9/11 (82%).

#### Analysis of Acute toxicity

All patients were evaluated weekly for acute GI and GU toxicities during the RT. Symptoms and treatment were recorded on the chart. We retrospectively reviewed these charts and graded acute GI and GU toxicities by the RTOG/EORTC acute radiation morbidity criteria [27]. Patients with rectal cancer were analyzed separately.

#### Statistical analysis

All statistical analyses were performed with a statistical software STATA Statistics/Data analysis (STATA Corp. 2001 Stata Statistical Software: Release 7.0 College Station, TX: Stata Corporation). The primary endpoints to be compared between both groups were incidence and severity of acute GI and GU toxicities during RT. The Chi-square frequencies test was used to verify the association between categorical variables and contingency tables. The Fisher's exact test was adopted in tables 2 × 2 when at least one expected frequency was lower than 5. The Student's t test was applied to verify association of numerical variables between the CRT and IMRT groups. A 5% significance level was considered for all statistical analyses.

## Results

The characteristics of CRT and IMRT patients are summarized in Table 1. All but tumor site distribution and RT goal are equally balanced in both groups.

The crude incidence of grade 2 acute GI (medication for diarrhea) was more frequent in the CRT group (65,2% *Vs *38,5%; *p *< 0.001) and absence of any GI symptoms (grade 0) was more frequently observed among patients treated with the IMRT technique (82.4% *Vs *17.6%; *p *< 0.001). Table 3 shows the crude incidence of acute GI toxicity according to RTOG/EORTC grading criteria.

**Table 3:** Crude incidence of acute GI toxicity in both groups according to RTOG/EORTC acute radiation morbidity criteria.

Grade	IMRT group (n = 65)	CRT group (n = 69)	*P *value
0	28 (43.1%)	6 (8.7%)	<0.001

1	12 (18.5%)	13 (18.8%)	0.955

2	25 (38.5%)	45 (65.2%)	0.002

3	0 (0%)	5 (7.2%)	0.058*

*Fisher's exact test

The crude incidence of acute GU complications was statistically similar in both groups (Table 4). Urinary symptoms not requiring medication (grade 1) were marginally more frequent among patients treated with IMRT (20% *Vs *8.7%, *p *= 0.06).

**Table 4:** Crude incidence of acute GU toxicity in both groups according to RTOG/EORTC acute radiation morbidity criteria.

Grade	IMRT group (n = 65)	CRT group (n = 69)	*P *value
0	40 (61.5%)	46 (66.6%)	0.54

1	13 (20%)	6 (8.7%)	0.06

2	12 (18.5%)	16 (23.5%)	0.50

3	0 (0%)	1 (1.5%)	> 0.99*

*Fisher's exact test

Patients with rectal cancer treated with IMRT presented a lower incidence of acute grade 2 (medication for diarrhea) GI toxicities (9.5% *Vs *65%; *p *< 0.01). Absence of any symptom (grade 0) was more frequently found in patients treated with IMRT (23.8% *Vs *5%; *p *= 0.077). Acute grade 1 GI toxicity was more frequent in patients from the IMRT group (66.6% *Vs *20%; p < 0.01) (Table 5). Crude incidence of acute GU toxicity was similar in both groups among patients with rectal cancer (Table 6).

**Table 5:** Crude incidence of acute GI toxicity in both groups according to RTOG/EORTC acute radiation morbidity criteria in patients with rectal cancer.

Grade	IMRT group (n = 21)	CRT group (n = 40)	*P *value
0	5 (23.8%)	2 (5%)	0.077

1	14 (66.6%)	8 (20%)	<0.001

2	2 (9.5%)	26 (65%)	<0.001

3	0 (0%)	4 (10%)	0.339*

*Fisher's exact test

**Table 6:** Crude incidence of acute GU toxicity in both groups according to RTOG/EORTC acute radiation morbidity criteria in patients with rectal cancer.

Grade	IMRT group (n = 21)	CRT group (n = 40)	*P *value
0	18 (85.7%)	26 (65%)	0.086

1	2 (9.5%)	4 (10%)	0.694

2	1 (4.8%)	9 (22.5%)	0.157

3	0 (0%)	1 (1.5%)	> 0.99*

*Fisher's exact test

## Discussion

Use of IMRT in the treatment of pelvic tumors has been increasing throughout the world for more than a decade. Our results of acute toxicity among patients in the IMRT group were presented at the 2009 Annual ASTRO meeting [28]. Many publications discuss the theoretical advantages of IMRT dose distribution and two complete revisions about its use in gynecological cancers have already been published [29,30]. Furthermore, there are several dosimetric studies that show reduction of dose delivered to the pelvic OARs with IMRT when compared with conventional or CRT techniques in the treatment of gynecological cancers [6-8,10,14,15], rectal cancer [5,11], anal canal cancer [9,13] and bladder cancer [12]. However, the main question is whether the dosimetric advantages of IMRT can lead to clinically relevant results when compared with non-modulated external beam RT.

Veldeman et al [31] made a systematic review of 41 comparative clinical studies with the use of IMRT that reported on overall survival, disease-specific survival, quality of life and/or treatment-induced toxicity, published prior to August 21, 2007. Concerning pelvic tumors, the authors did not find any prospective study that compares IMRT with non-IMRT technique. Furthermore, no study about overall survival, disease-specific survival or quality of life had been published until then. These authors identified three comparative case series for gynecological malignancies that significantly showed lower rates of acute GI toxicity [16,17,24], one with less chronic GI toxicity [18], one with less hematological side effects [23] and one with lower acute GU toxicities [24] in patients treated by pelvic IMRT in comparison to those treated by non-IMRT techniques. For anal canal cancer, they included just one non-comparative case series with 17 patients that showed no grade 3 or higher acute non-hematological toxic effects or treatment breaks attributable to GI or skin toxicity [9].

Other clinical studies have also been published about use of IMRT in gynecological cancers [19,20,22], and [25], in rectal cancer [21] and in bladder cancer [12]. All these studies showed a lower rate of radiation-induction toxicity with IMRT.

Considering evidence-based medicine, multi-institutional prospective clinical trials are important to corroborate the real benefit of IMRT in the treatment of pelvic tumors. The Radiation Therapy Oncology Group (RTOG) is conducting a prospective phase II study of IMRT for postoperative patients with either endometrial or cervical carcinoma with or without chemotherapy (RTOG 0418) and the Tata Memorial Hospital, in Mumbai, India, is conducting the only ongoing prospective phase II randomized trial comparing conventional RT versus IMRT in the treatment of cervical cancer. Results of these two trials will contribute to assess the benefits and risks of IMRT in patients with gynecologic tumors.

The most important result from our series was the lower incidence of medication for diarrhea (grade 2) among patients treated with IMRT. Diarrhea is a very uncomfortable symptom and can cause dehydration and malabsorption of vitamins, lactose, and bile acids [4]. Another important finding was the higher absence of GI symptoms (grade 0) in IMRT group (43.1% versus 8.7%; *p *< 0.001). The possibility of offering a greater opportunity to avoid GI symptoms to patients under RT treatment is a considerable advantage for IMRT. Because use of CT is now well established for treatment of some pelvic tumors sites, such as the rectum, cervix, anal canal and bladder, IMRT can be very useful to reduce the acute toxicities potentialized by CT since it not only improves delivery of CT but also potentially provides conditions for CT dose escalation.

In our series, use of IMRT did not reduce acute GU toxicities. The incidence of acute grade 1 GU side effects was marginally more frequent in the IMRT group (20% versus 8.7%; *p *= 0.06) as shown in table 4. As grade 1 acute GU radiation morbidity is defined by RTOG/EORTC criteria as "Frequency of urination or nocturia twice pretreatment habit and dysuria or urgency not requiring medication" [27], this difference is not important in clinical practice and definition of this grade could be subjective, as the information collected was based on physician's notes in patient's charts.

Our results of lower acute GI toxicity in the IMRT group and similar acute GU toxicity in both groups were like those reported by the Mundt et al. [17] through a comparative case series for women with gynecological malignancies. They reported on grade 2 acute GI toxicity less common in the IMRT group than in the conventional RT (60% vs. 91%; *p *= 0.002) and grade 2 GU toxicity not statistically significant (10% vs. 20%; *p *= 0.22).

Due to the relatively greater number of patients with rectal cancer in the CRT group and that almost all had been treated by combined CT with capecitabine (98% in the CRT group and 86% in the IMRT group), we performed a separate analysis of these patients. Absence of GI symptoms (grade 0) was greater in IMRT group (23.8% versus 5%; *p *= 0.07), as shown in table 5. Medication for diarrhea (grade 2) was significantly lower in the IMRT group (9.5% versus 65%; p < 0.001). Considering that capecitabine alone can also cause diarrhea and increase radiosensitivity, this finding is considerably positive in favor of IMRT. Curiously, grade 1 acute GI toxicity was more often found among patients treated by IMRT. Because grade 1 acute GI side effects are described by RTOG/EORTC criteria as "increased frequency or change in quality of bowel habits nor requiring medication or rectal discomfort not requiring analgesics" [27], this finding is not relevant in the clinical practice and these symptoms are a subjective endpoint.

No difference was observed in crude incidence of acute GU toxicity in patients with rectal cancer treated with CRT or IMRT technique (Table 6) as we also observed when all patients with other primary tumor sites are considered (Table 4). These findings suggest that the bladder is less sensitive to reductions in volume irradiated than the small bowel, especially when the total dose is up to 50 Gy. We also must consider that the low number of events could have limited the statistical power of this analysis.

**Figure 1:**
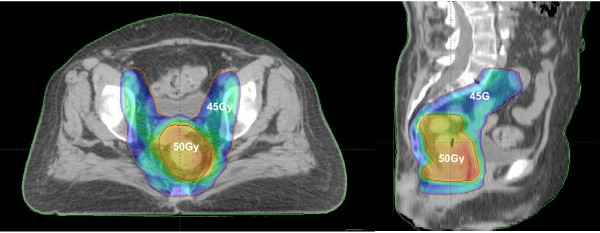
**Dose distributions with SIB-IMRT strategy at lymph node regions and primary tumor site in a patient with low rectal cancer, to receive 45 Gy (blue painting) and 50 Gy (orange painting), respectively, in 25 daily fractions**.

Another advantage of IMRT is the possibility to deliver a different level of daily dose to the distinct target volumes. In our Institution, we routinely treat patients with rectal cancer with preoperative RT concomitant to CT and due to the lesser probability of small bowel toxicity with IMRT, all patients are nowadays treated with this technique using synchronous integrated boost (SIB) strategy to deliver 50 Gy (2 Gy/fraction) to the gross primary tumor while simultaneously delivering 45 Gy (1.8 Gy/fraction) to the regional lymph nodes and areas of risk for harboring microscopic disease (Figure 1). There is one ongoing prospective fase II trial using preoperative SIB-IMRT strategy and capecitabine for treatment of locally advanced rectal cancer [21]. In this study, a total dose of 55 Gy (2.2 Gy/fractions) is delivered to the primary tumor and of 45 Gy (1.8 Gy/fractions) to the lymph nodes regions in 25 fractions. The preliminary results already published, with only eight patients showed an impressive pathologic complete response rate of 38% with minimal toxicity. These results warrant further evaluation in future larger cooperative and prospective phase II or phase III trials.

In conclusion, this retrospective and comparative case series showed that use of the IMRT technique to treat pelvic tumors reduced the frequency and severity of GI symptoms and the need of medication for diarrhea in comparison to the CRT technique, but did not reduce incidence of acute GU toxicities. For rectal cancer patients these benefits were also observed, even with concomitant CT. For these reasons, the IMRT technique, when available, should be considered to treat pelvic tumors whenever the lymph nodes and primary tumor sites must be irradiated.

## List of abbreviation

ASTRO: Americal Society of Therapeutic Radiation Oncology; CRT: Conformal Radiation Therapy; CT: Chemotherapy; CTV: Clinical Target Volume; EORTC: European Organization on Radiation Therapy Consortium; GI: Gastrointestinal; GU: Genitourinary; ICRU: International Comission on Radiation Unit and Mensurements; IMRT: Intensity Modulated Radiation Therapy; OAR: Organ at Risk; PTV: Planning Target Volume; RT: Radiation Therapy; RTOG: Radiation Therapy Oncology Group; SIB: Simultaneous Integrated Boost;

## Competing interests

The authors of the present manuscript (R Ferrigno, A Santos, LC Martins, E Weltman, M Chen, R Sakuraba, CP Lopes, VD Gonçalves, and JC da Cruz) declare that they have no competing interests.

## Authors' contributions

RF carried out the patients' data from their charts and wrote the manuscript.

AS separated and organized the patient's charts.

LCM helped to verify the literature data about IMRT.

EW participated in the identification and classification of acute gastrointestinal toxicities.

MJC participated in the identification and classification of acute gentitourinary toxicities.

RS performed the statistical analysis.

CPL helped the statistical analysis calculation.

JCC participated in the figures configuration and helped to write the manuscript.

All authors read and approved the final manuscript.
